# DNA methylation differences in noncoding regions in ER negative breast tumors between Black and White women

**DOI:** 10.3389/fonc.2023.1167815

**Published:** 2023-05-24

**Authors:** Jianhong Chen, Michael J. Higgins, Qiang Hu, Thaer Khoury, Song Liu, Christine B. Ambrosone, Zhihong Gong

**Affiliations:** ^1^ Department of Cancer Prevention and Control, Roswell Park Comprehensive Cancer Center, Buffalo, NY, United States; ^2^ Department of Molecular and Cellular Biology, Roswell Park Comprehensive Cancer Center, Buffalo, NY, United States; ^3^ Department of Biostatistics and Bioinformatics, Roswell Park Comprehensive Cancer Center, Buffalo, NY, United States; ^4^ Department of Pathology & Laboratory Medicine, Roswell Park Comprehensive Cancer Center, Buffalo, NY, United States

**Keywords:** breast cancer, DNA methylation, noncoding regions, ER negative tumor, Black and White women

## Abstract

**Introduction:**

Incidence of estrogen receptor (ER)-negative breast cancer, an aggressive tumor subtype associated with worse prognosis, is higher among African American/Black women than other US racial and ethnic groups. The reasons for this disparity remain poorly understood but may be partially explained by differences in the epigenetic landscape.

**Methods:**

We previously conducted genome-wide DNA methylation profiling of ER- breast tumors from Black and White women and identified a large number of differentially methylated loci (DML) by race. Our initial analysis focused on DML mapping to protein-coding genes. In this study, motivated by increasing appreciation for the biological importance of the non-protein coding genome, we focused on 96 DMLs mapping to intergenic and noncoding RNA regions, using paired Illumina Infinium Human Methylation 450K array and RNA-seq data to assess the relationship between CpG methylation and RNA expression of genes located up to 1Mb away from the CpG site.

**Results:**

Twenty-three (23) DMLs were significantly correlated with the expression of 36 genes (FDR<0.05), with some DMLs associated with the expression of single gene and others associated with more than one gene. One DML (cg20401567), hypermethylated in ER- tumors from Black versus White women, mapped to a putative enhancer/super-enhancer element located 1.3 Kb downstream of *HOXB2*. Increased methylation at this CpG correlated with decreased expression of *HOXB2* (Rho=-0.74, FDR<0.001) and other *HOXB/HOXB-AS* genes. Analysis of an independent set of 207 ER- breast cancers from TCGA similarly confirmed hypermethylation at cg20401567 and reduced *HOXB2* expression in tumors from Black versus White women (Rho=-0.75, FDR<0.001).

**Discussion:**

Our findings indicate that epigenetic differences in ER- tumors between Black and White women are linked to altered gene expression and may hold functional significance in breast cancer pathogenesis.

## 1 Introduction

Evidence from both epidemiological and large-scale consortium studies supports the hypothesis that estrogen receptor positive (ER+) and negative (ER-) breast tumors derive from distinct etiologic pathways (1, 2). Compared to women diagnosed with ER+ breast cancer, those with ER- tumors in general have a poor prognosis, partly due to their aggressive phenotype and the lack of targeted therapy. ER- breast cancer is more common among Black women than White women (3, 4), but distinct reasons for these disparities remain to be elucidated.

DNA methylation, a major epigenetic mechanism, plays crucial roles in hormone-induced differentiation and tissue remodeling of the mammary gland through the life course (5). Aberrant DNA methylation patterns in breast cancer have been widely observed, and could contribute to differences in breast cancer risk between Black and White women (6–9). Studies have reported that hypermethylation at promoter regions of tumor suppressor genes, such as *RASSF1A* and *CDH13*, was inversely associated with gene expression, and that expression levels of these genes were lower in ER- tumors from Black women than those in White women, representing potential underlying tumor biological mechanisms explaining breast cancer racial disparities (10, 11). In addition, our previous research identified differentially methylated loci (DML) by tumor ER subtype and between races, with more DMLs by tumor ER subtype among Black women than that in White women; the number of race-related DMLs identified in ER- tumors were almost twice as those identified in ER+ tumors (6, 12).

Together, aberrant DNA methylation patterns have been used to dissect breast cancer risk by tumor subtype and racial groups, with potential diagnostic and prognostic applications (13, 14). However, previous studies have mainly focused on DNA methylation alterations associated with protein coding genes, with little attention on non-protein coding regions, which constitute more than 98% of the whole human genome.

It has long been known that a large portion of aberrant DMLs in breast cancer is located in intergenic regions (15), which constitute about 50% of the human genome (16). In a genome-wide expression-methylation quantitative loci (emQTL) analysis, Fleischer et al. reported several hundred regulatory elements not associated with protein coding genes whose methylation alterations were associated with different breast cancer lineages (17). Enhancers are critical *cis*-regulatory elements within non-coding regions, which contain the majority of cancer-associated variants based on genome-wide association studies (18). Studies have also revealed that enhancers are the most consistently differentially methylated regions and that their differential methylation is in a cell-type-specific manner, indicating the importance of enhancer methylation for epigenetic regulation of tumorigenesis (19). The underlying mechanisms could be that certain DMLs overlapping with enhancers can regulate tumor-associated genes and pathways, subsequently playing important roles in cancer (17, 20, 21). In addition, focused on small noncoding RNAs, microRNAs (miRNAs), we previously found that several hundred DMLs that mapped to miRNA genes were differentially methylated by tumor ER subtype and between Black and White women, and that their methylation levels were significantly correlated with corresponding miRNA gene expression (22). In summary, these studies highlight the importance of DMLs occurring within non-protein coding regions in relation to risk of breast cancer, especially their potential roles in explaining breast cancer racial disparities.

As described previously, we conducted genome-wide DNA methylation profiling on breast tumor tissue samples obtained from participants in the Women’s Circle of Health Study (WCHS), a case-control study designed to investigate risk factors for aggressive breast cancer in Black and White women (12, 23). Our initial analysis focused on DMLs mapping within or near protein-coding genes, and revealed that a key pro-luminal transcription factor, *FOXA1*, was hypermethylated and repressed in tumors from Black women compared to White women (12). Herein, we focus on DMLs mapped to non-protein coding regions due to their biological importance and limited research in the area.

Motivated by the biological significance of the noncoding genome and our research interests in understanding the higher risk of ER- breast cancer in Black compared to White women, we aimed to identify DMLs by race within ER- tumors, with a focus on DMLs located in intergenic and noncoding RNA genomic regions. In addition, we integrated both DNA methylation (Illumina Infinium 450K array) and gene expression (RNA sequencing) data to examine whether DMLs were associated with altered gene expression. Our results were then validated using The Cancer Genome Altas (TCGA) dataset. We further investigated the epigenomic context and molecular features of the top DMLs confirmed in both our and TCGA datasets to determine their regulatory potential and biological functions.

## 2 Materials and methods

### 2.1 Study population and tissue samples

Data and breast tumor tissue samples were from participants enrolled in the WCHS. Details on the study design and participant recruitment have been described previously (12). The study protocol was approved by Institutional Review Boards at all participating institutes. In-home interviews were conducted to obtain data on known and suspected risk factors for breast cancer. As part of the informed consent, >95% participants signed a release for their pathology reports and archived specimens in form of formalin-fixed, paraffin-embedded (FFPE) tumor blocks, which were obtained from the pathology departments of the treating hospitals. Data on tumor pathological features, including ER status, were extracted from the pathology reports.

### 2.2 DNA extraction, DNA methylation profiling, and data processing

DNA was extracted from FFPE tumor tissues among 694 women enrolled in the WCHS (**Table S1**) as previously described (12). Briefly, FFPE samples were deparaffinized in xylene, lysed, and incubated at 56°C with constant rotation until completely digested. Lysates were then heated at 70°C for 20min to inactivate the Proteinase K and stored at 4°C. DNA from a 5ul aliquot of FFPE lysate was purified using the DNA Clean & Concentrator-5 kit. Genome-wide DNA methylation analysis was carried out at Roswell Park Genomics Shared Resource using the Illumina Infinium HumanMethylation450 BeadChip platform, which interrogates > 485,000 CpG dinucleotides per sample at single-nucleotide resolution and covers 99% of RefSeq genes. To minimize the impact of batch effects, DNA samples from tumors were randomized on plates according to age, race, and ER status. The raw intensities from the array were extracted using GenomeStudio, and the data summarized into BeadStudio IDAT files and processed by the minfi R package. The methylation level of each CpG site, calculated as a β value, ranged between (0, 1), with 0 for absent methylation and 1 for complete methylation. In brief, the 450K array data were subjected to rigorous sample and locus specific quality control criteria, SWAN normalization, and correction for batch effects using the ComBat algorithm (24). Low quality probes (probes with detection p value > 0.05 in more than half of samples) and samples with poor detection p values (samples with detection p values < 1 x 10^–5^ at more than 75% of CpG loci) were removed using the IMA package (25). We used Bowtie 2 for sequence alignment (26). Probes known to map ambiguously, exhibiting cross-reactivity, and that contain single nucleotide polymorphisms were also removed, leaving the final dataset containing 276,108 CpG loci in 694 tumor samples for final analyses (27, 28).

### 2.3 Data analysis pipeline

**Figure 1:**
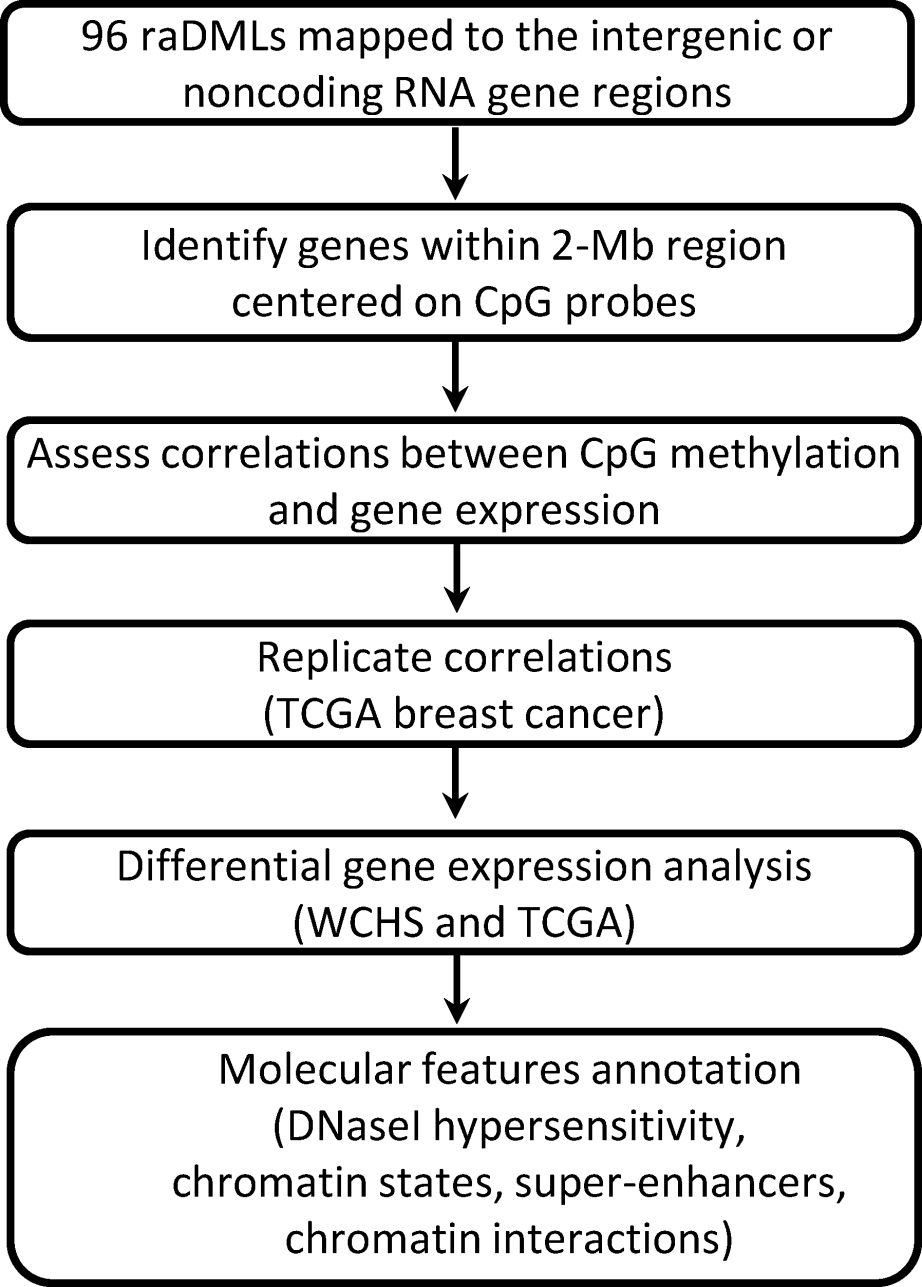
Study flowchart.

Unlike previous studies that focused primarily on CpGs located near a protein-coding gene, we investigated CpGs mapped to intergenic regions or noncoding RNAs in proximity, and validated results in the independent TCGA breast cancer cohort. We performed functional annotations for top CpGs to determine their potential roles in breast cancer racial disparity by exploring their integrative biologic context from multiple genome and epigenome databases, their associations with target gene expression, and differential gene expression patterns between Black and White women, as shown in **Figure 1**, the overall workflow.

#### 2.3.1 Differentially methylated loci in ER- tumors between Black and White women within noncoding regions

Differences in DNA methylation β-values for each probe were evaluated in ER- tumors by race. The Wilcoxon rank-sum test was used to evaluate the statistical significance for each probe in the comparison. To adjust for multiple comparisons, the false discovery rate (FDR) was computed using the Benjamini and Hochberg approach. Differentially methylated loci in ER- tumors between Black and White women (raDMLs) were defined as CpGs with an absolute mean β value difference (|delta β|) at least 0.10 between two race groups and *FDR*-adjusted p value <0.05. All analyses were performed using R package.

#### 2.3.2 Association between DNA methylation and gene expression

Genes located within a 2 Mb window centered on a CpG site were considered as potential regulatory targets of DNA methylation (29–31). Thus, for each raDML, we adopted a window width of 1Mb on either side of the CpG and assessed the correlations between CpG methylation level and relative RNA expression level of genes within this window. For each CpG/gene pair, the Spearman correlation between DNA methylation (β values) and relative gene expression levels (log counts per million, log CPM) was assessed. An independent collection of 50 fresh frozen breast tumor samples from Roswell Park Pathology Network Shared Resource (PNSR) was used for RNA extraction and relative gene expression analysis as previously described (6, 12). An association was considered significant if *FDR*-adjusted p value <0.05. Only significant associations were included for further functional annotations, which were then validated by repeating the same analysis using the TCGA breast cancer cohort.

#### 2.3.3 Differential gene expression analysis

Genes with significant correlation between DNA methylation and relative gene expression (CpG/gene pairs) were analyzed for differential expression between Black and White women in ER- tumors using DESeq2 (32). P values were calculated using linear regression function of DESeq2, with adjustment of age at diagnosis and corrections for multiple testing.

#### 2.3.4 TCGA data processing and analysis

To validate our findings, level 3 Illumina HM450 methylation data, Hiseq2 gene expression data, demographic and related clinical features (e.g., age, race, ER status, and tumor stage) of the 1,097 cases included in the TCGA breast cancer cohort were downloaded from the publicly available FireBrowse database (http://firebrowse.org). Validation of race-related methylation difference, association between methylation and associated gene expression, and differential gene expression analysis by race in ER- tumors were conducted following the same pipeline as described above.

#### 2.3.5 Molecular feature annotation

For CpGs showing statistically significant correlation with gene expression confirmed in both our and TCGA dataset, we further investigated their epigenomic context and determined their regulatory potential. Specifically, the regional chromatin landscape in proximity to each CpG site was investigated using multiple publicly available databases. Chromatin state annotations were extracted from the Roadmap Epigenomics ChromHMM on ENCODE (E027 and E119). To facilitate functional interpretation, we focused on six ChromHMM states, including TssA (Active TSS), TssAFlnk (Flanking Active TSS), TssBiv (Bivalent/Poised TSS), EnhG (Genic enhancers), Enh (Enhancers), and EnhBiv (Bivalent Enhancer). DNaseI hypersensitive sites indicative of an open chromatin structure with potential transcriptional activity were similarly identified from the Roadmap/ENCODE reference epigenomes. GeneHancer module under the GeneCards Suite comprises a large collection of enhancer- gene association, which was used to annotate enhancer regions and their associated genes (33). We further inferred super-enhancer regions using a catalogue of super-enhancers in HMEC and MCF-7 cell lines published elsewhere (34). The UCSC Genome Browser was used to visualize genomic features centered on these CpGs. A circos plot summarizing global molecular features of these CpGs was generated using OmicCircos version 1.24.0. All statistical analyses were performed using the R statistical software.

## 3 Results

### 3.1 DMLs mapped within non-protein coding regions in ER- tumors between Black and White women

In our previous analysis (12), we identified a total of 396 raDMLs that exhibited significant differential methylation between Black and White women within the ER- breast cancer group. Of the 396 raDMLs, 276 CpGs were assigned with at least one protein-coding gene based on the Illumina self-manifestation file (https://support.illumina.com/downloads/infinium_humanmethylation450_product_files.html), leaving 120 CpGs uncharacterized. We further excluded 24 CpGs that were mapped in the protein-coding region based on an updated Ensemble gene annotation file (Ensemble Gene 104, http://www.ensembl.org/). In the end, the remaining 96 raDMLs mapped to intergenic regions or noncoding RNA regions, which were the focus of the current study (**Table S2**). Validation of observed methylation differences by race at each CpG site was then conducted using the TCGA breast cancer cohort.

Among the 96 raDMLs within non-protein coding regions in ER- tumors between Black and White women, 59 of these CpGs were located in intergenic regions and the remaining 37 CpGs mapped to at least one non-coding RNA gene. Consistent with our previous findings on protein-coding genes (12), there were more hypomethylated CpGs within the non-protein coding genome. Specifically, of the 96 raDMLs, 58 CpGs were hypomethylated and 38 CpGs were hypermethylated in ER- tumors from Black compared to White women.

Out of the 96 raDMLs, data on 59 CpGs were available in the TCGA dataset. Except for one CpG site (dot in red), all other probes showed consistent direction of methylation change by race (dot in black) in the TCGA dataset (**Figure S1**).

### 3.2 Associations between DNA methylation and gene expression

The 96 raDMLs were found to be associated with 1,998 unique genes, which correspond to a total of 2,357 unique CpG/gene pairs. Using previously described DNA methylation and RNA-seq data from analysis of an independent group of 50 fresh frozen breast tumor samples (6, 12), Spearman correlation between DNA methylation and gene expression was assessed for each CpG/gene pair. Among the 2,357 unique CpG/gene pairs, analysis identified significant correlations for 39 CpG/gene pairs, corresponding to 23 unique CpGs and 36 unique coding or noncoding RNA genes (FDR-adjusted p value < 0.05, **Table S3**). Of the 36 unique gene/RNAs, we found six long noncoding RNAs (lncRNAs), including three antisense lncRNAs (*SOX9-AS1*, *HOXB-AS1*, and *HOXB-AS3*), a long intergenic noncoding RNA (*LINC01152*), and two uncharacterized lncRNAs (*LOC102723517*, *LOC283335*). Overall, most CpG/gene pairs (25/39, 64.1%) exhibited positive correlations between methylation and gene expression, while 14/39 (35.9%) pairs showed negative correlations.

**Table 1** listed the top 10 CpG/gene pairs, including 5 unique CpGs with associated genes or lncRNAs. Notably, we identified several CpG sites at which methylation was correlated with the expression of multiple genes. Cg20401567 is the top CpG site in proximity to the *HOXB* gene cluster, with its methylation level inversely, highly associated with the expression levels of multiple members of the *HOXB* gene family, including *HOXB2* (correlation coefficient, rho=-0.74) and *HOXB3* (rho=-0.62). Previously, we reported methylation level at cg04932551, a raDML within the gene body of *FOXA1*, inversely correlated with *FOXA1* expression (12). In this study, we discovered another CpG, cg12212453 located at 5 kb downstream of *FOXA1*, whose methylation level was also strongly, inversely correlated with *FOXA1* gene expression (rho=-0.67). We identified a novel CpG, cg05322837, whose methylation level was correlated with expression of two lncRNAs, *LOC102723517* and *LINC01152* (rho=0.65 and 0.58, respectively), and a Solute Carrier Family 39 gene, *SLC39A11* (rho=-0.61). In addition, we found the methylation level of cg05199874 was positively correlated with expression of multiple genes, including the signal peptide-CUB-EGF domain-containing protein 2 (*SCUBE2*), a novel tumor suppressor gene, which showed inhibitory roles in breast tumor invasion and migration through concerted activities with *FOXA1* (35, 36). Moreover, methylation level of cg12821539 was positively correlated with expression of *ZIC5* (rho=0.62), which has been implicated as oncogenes in some cancers (37).

**Table 1:** Top ten CpG/gene pairs based on methylation-gene expression correlation analysis.

CpG ^a^	Gene ^b^	Distance (Kb) ^c^	deltaBeta^d^	rho^e^	FDR^f^
cg20401567	*HOXB2*	3.3	0.12	-0.74	2.90E-06
	*HOXB3*	-6.7	0.12	-0.62	7.40E-04
cg12212453	*FOXA1*	4.8	0.12	-0.67	1.10E-04
cg05322837	*LOC102723517*	117.5	0.13	0.65	2.30E-04
	*LINC01152*	121	0.13	0.58	3.00E-03
	*SLC39A11*	-494.3	0.13	-0.61	8.00E-04
cg05199874	*TMEM41B*	32.1	-0.13	0.59	2.50E-03
	*RNF141*	-910	-0.13	0.59	2.50E-03
	*SCUBE2*	582.1	-0.13	0.55	8.30E-03
cg12821539	*ZIC5*	27.6	0.11	0.62	7.40E-04

**^a^** Illumina 450K CpG probe.

**^b^** RefSeq genes located ≤1 Mb away from CpG.

**^c^** Genomic distance (kb) between CpG probe and transcriptional start site of the indicated gene.

**^d^** DNA methylation difference (delta Beta) at indicated CpGs comparing Black vs. White women.

**^e^** Spearman correlation coefficient (rho).

**^f^** False discovery rate (FDR) q value derived from correlation between beta values and RNA expression levels for an indicated CpG/gene pair.

*HOXB* gene family, *FOXA1*, *SLC39A11*, and *SCUBE2* were found to play well-established roles in breast cancer development (12, 35, 38), and thus were the focus on our further analysis.

**Figure 2:**
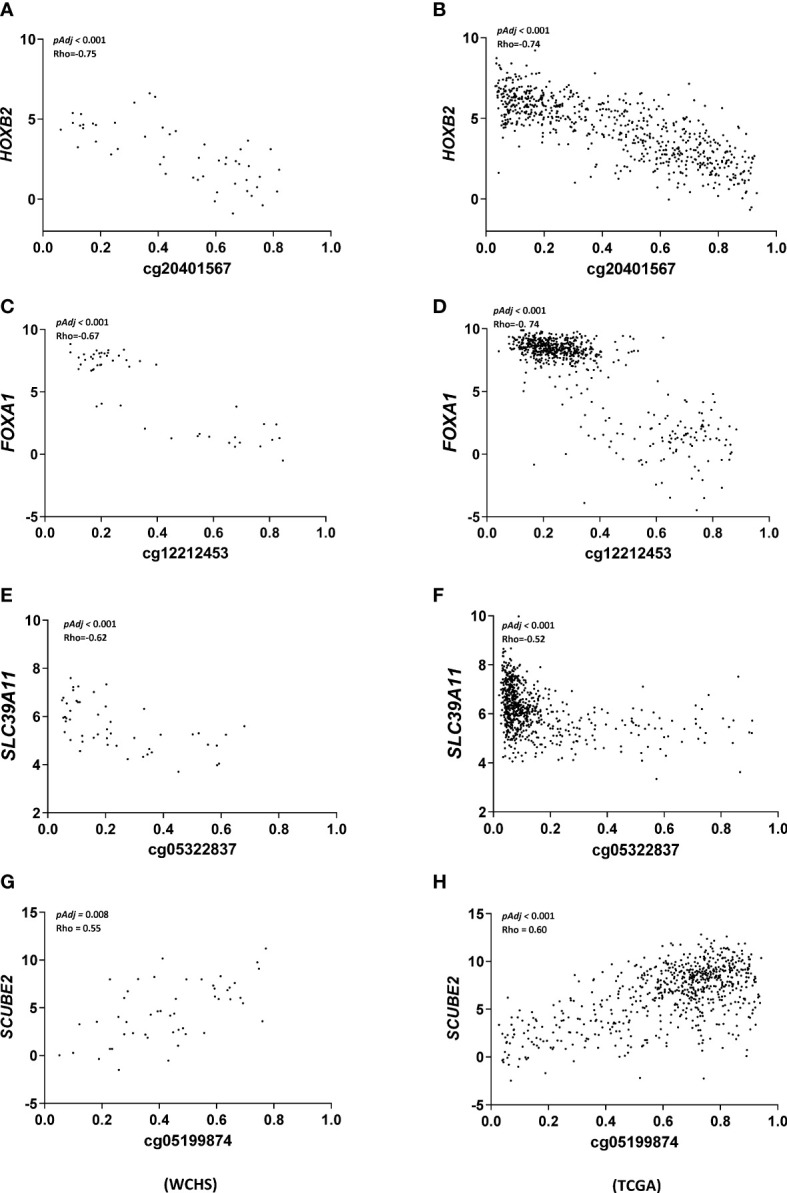
Scatter plot summarizing correlation analysis of top ranked CpG/gene pairs. X-axis denotes CpG site methylation levels in b value and Y-axis denotes relative gene expression of the associated genes in logCPM. For each pair [cg20401567 and HOXB2 **(A, B)**, cg12212453 and FOXA1 **(C, D)**, cg05322837 and SLC39A11 **(E, F)**, and cg05199874 and SCUBE2 **(G, H)**], Spearman correlation was used to test the relationship between DNA methylation and gene expression in WCHS (left panel) and the TCGA (right panel) breast cancer cohort with p values and correlation coefficients labeled in the inlet.

We further validated these findings by repeating the correlation analysis in the TCGA breast cancer cohort. Limited by data availability of HM450 DNA methylation and Hiseq gene expression in the TCGA breast cancer cohort, only 28 out of the 39 significant CpG/gene pairs identified in our analysis were available and thus included in the validation analysis. Nevertheless, 20 out of these 28 CpG/gene pairs were validated with respect to the magnitude and direction of the correlation coefficient and all reached statistical significance (**Table S4**). The remaining 8 CpG/gene pairs did not reach statistical significance in the TCGA cohort. As shown in **Figure 2**, we further showed in scatter plots for the top four highly correlated CpG/gene pairs that exhibited the most consistent correlation between methylation and gene expression in both WCHS and TCGA breast cancer cohort, respectively.

### 3.3 Molecular features accounting for regulatory effects of aberrant DNA methylation

**Figure 3:**
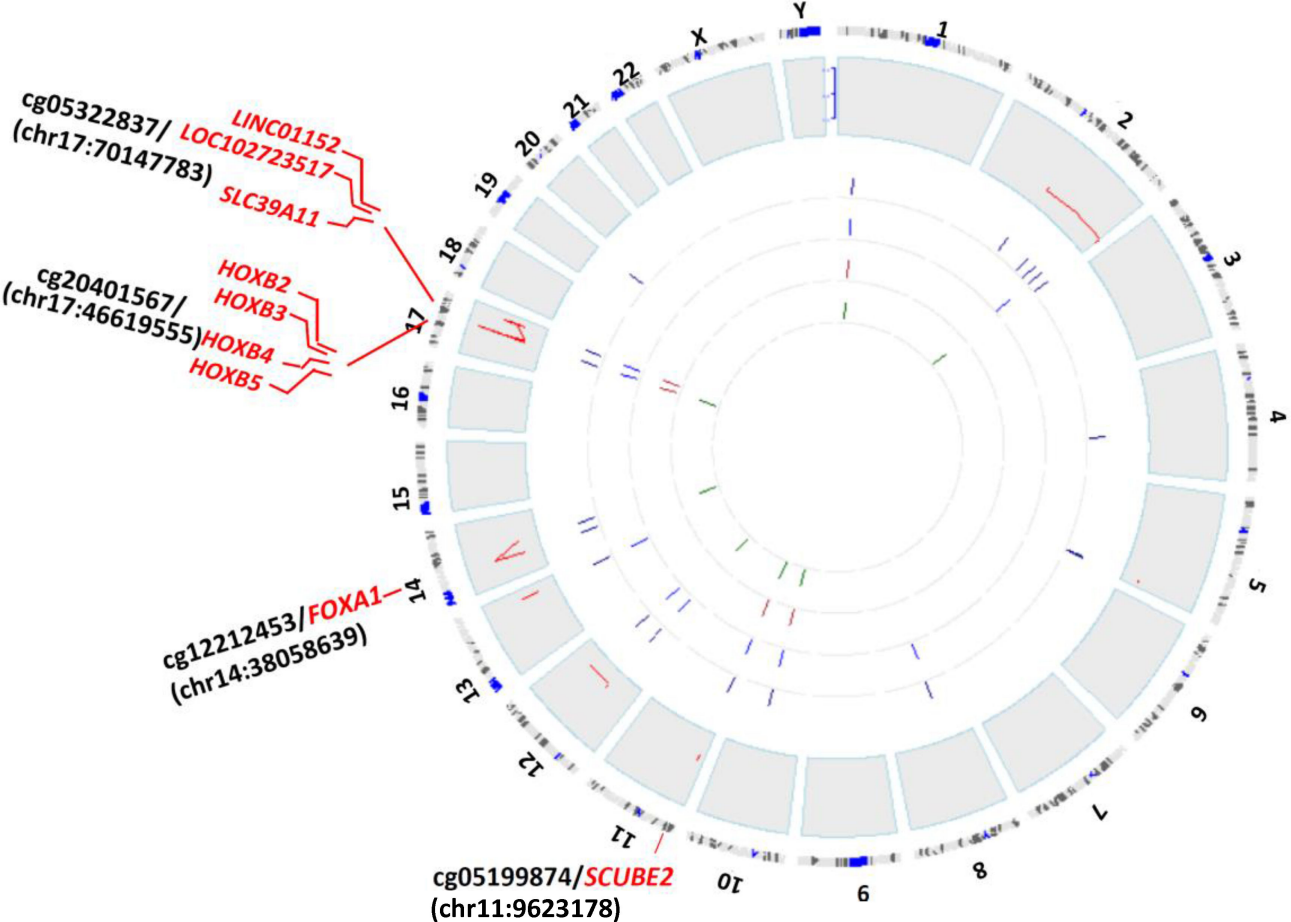
Visualization of genomic features of the 23 CpG sites. OmicCircos plot showed overall landscape of these CpG sites. Six tracks were displayed, from outside to inside: chromosome cytobands in black and blue, –log10 transformation of Spearman correlation p values (<0.05) between methylation and associated gene expression in red, the 23 CpG sites in navy blue, ChromHMM features of promoter and enhancer annotation in blue, hypersensitive DNase I sites annotation in brown, and super enhancer annotation of these probes in dark green. The top four CpGs and their associated gene (genes) were labeled in red.

**Figure 4:**
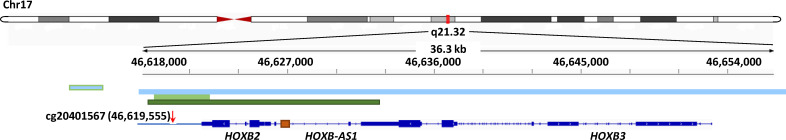
Relative genomic positions of cg20401567, *cis*-elements, *HOXB2*, *HOXB3*, and *HOXB-AS1* on chromosome 17. The chromosome ideogram was shown on top, with a region spanning this locus highlighted in red. Cg20401567, *cis*-elements, *HOXB* family members, and *HOXB-AS1* in this locus were displayed at the bottom of the diagram, with genomic coordinates labeled in the middle. *Cis*-element annotations from various resources around Cg20401567 were labeled, including two promoter/enhancers based on GeneCards (light blue), an enhancer based on ChromHMM (light green), and a super enhancer based on Hnisz D et al. (34) (dark green). In addition, at bottom of the plot, a promoter upstream of *HOXB2* was displayed (orange).

Chromatin architecture in which DNA methylation occurs provide important clues as to how DNA methylation alterations mediate their effects in disease predisposition. For each of the 23 unique CpGs (out of the 39 CpG/gene pairs) exhibiting significant correlations with its paired gene, we annotated their molecular features globally, including genomic position, ChromHMM state, DNaseI hypersensitive sites, enhancer and super enhancer sites, and their correlations between CpG methylation and RNA expression. As shown in **Figure 3**, cg20401567, cg12211453, cg05322837, and cg05199874, paired with the *HOXB* gene cluster, *FOXA1, SLC39A11*, and *SCUBE2*, respectively, were highly enriched with candidate *cis-*regulatory elements (cCREs), characterized by hypersensitive DNase I sites, promoter/enhancer-related ChromHMM segments, H3K4m1/2/3 and H3K27ac histone modification, implicating them as subjects of intensive gene expression regulation through DNA methylation modifications. As we showed in supplementary **Figure S2**, the Genome Browser plot exhibits regional genomic features of enrichment of DNase I hypersensitive site, ChromHMM segments implicating promoter, and histone marks predictive of open chromatin region around the genomic position of cg20401567. Consistent with our results, the region around cg20401567 includes not only *HOXB* gene family members, but also *HOXB-AS* genes. The relative position of *cis*-elements, cg20401567, *HOXB2*, *HOXB3*, and *HOXB-AS1* on chromosome 17 was shown in **Figure 4**. Enhancer, super enhancer, and promoter annotations are obtained from various resources and their genomic positions are overlapped with each other. In addition to enrichment of *cis*-elements, we also observed high DNA sequence conservation across vertebrates for this region, indicative of important biological functions.

### 3.4 Expression differences on raDML-associated genes in ER- tumors between Black and White women

**Figure 5:**
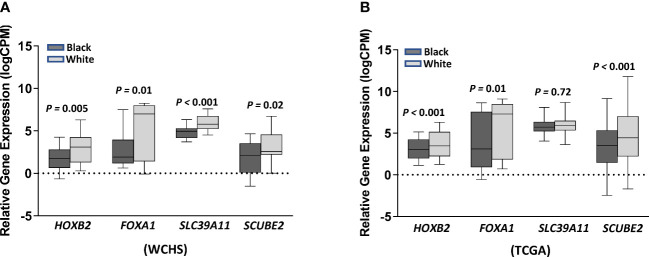
Gene expression levels of *HOXB2, FOXA1*, *SLC39A11*, and *SCUBE2* in ER- tumors between Black and White women from the WCHS cohort **(A)** and the TCGA **(B)** breast cancer cohort. Relative gene expression levels (logCPM) were presented as mean± standard errors (SE). Gene expression differences between Black and White women were tested using DESeq2 after adjustment of age at diagnosis, with adjusted p values labeled on top. Black and grey denotes relative gene expression levels in Black and in White women, respectively.

We further investigated whether the top CpG-correlated genes were differentially expressed in tumor tissues between Black and White women within the WCHS and TCGA breast cancer cohort. Except for *SLC39A11*, the other three genes (*HOXB2*, *FOXA1*, and *SCUBE2*) exhibited significantly lower expression levels in tumors from Black versus White women in both study cohorts after adjusting for age at diagnosis (**Figure 5**), which were consistent with observed CpG-gene expression correlation, with higher methylation at cg20401567 and cg12212453 (paired with *HOXB2* and *FOXA1*, respectively), and lower methylation at cg05199874 (paired with *SCUBE2*) in Black relative to White women. These results suggested that differential DNA methylation between races and their altered gene expression may contribute to breast cancer racial differences.

## 4 Discussion

Noncoding DNA regions comprise more than 98% of the human genome and play key roles in regulating gene expression through various mechanisms (39). For instance, aberrant methylation in intergenic regions, such as enhancers, has been shown to be associated with altered expression of neighboring genes, including those involved in cell cycle processes, lymphocyte activation and apoptosis (17, 40). Very few prior studies have focused on identifying differentially methylated features, especially in noncoding genome regions, in breast tumors between Black and White women, which may contribute to race-related differential predisposition to aggressive tumor subtypes (6, 10–12). To our knowledge, we are one of the few to globally investigate the role of noncoding region DNA methylation patterns in relation to breast cancer racial disparities (22, 41).

Intergenic regions and lncRNA genes, which comprise most of the non-protein coding genome, are enriched in epigenetic modifications, and have elicited great efforts to systematically annotate the regulatory elements existing within these regions. In this study, we identified several CpGs located within the noncoding genome that were differentially methylated in ER- tumors between races, with some CpGs highly correlated with expression of specific protein coding and/or lncRNA genes. Using multiple publicly available epigenome databases, we further characterized molecular features of these CpGs, such as active histone modifications, chromatin accessibility, and enhancer/super-enhancer sequences. We found that most of the 96 raDMLs mapping to intergenic regions or lncRNA genes were in at least one of these functional elements, with some CpGs enriched by multiple functional elements. The rich content of putative regulatory elements located at intergenic CpGs suggests active regulation of transcriptional activity through DNA methylation modification in these regions. Consistent with our observation, Kamalakaran et al. reported that among featured DNA methylation sites efficiently distinguishing the five major breast cancer subtypes, 70% are within non-protein coding regions, while only 30% of the sites mapped to genes encoding proteins (15). It should be noted, that the high occurrence of these functional elements within intergenic noncoding regions might be also due to the fact that intergenic CpG loci of the original HM450 chip design were preferentially selected toward biologically significant/informative sites, DNase hypersensitive sites, and differentially methylated regions (42, 43). Nevertheless, our findings that these CpGs were differentially methylated between two racial groups and associated with altered gene expression, support their potential roles contributing to breast cancer differences between Black and White women.

One of the interesting observations is that aberrant DNA methylation occurring in CpG islands or within gene body generally correlates with expression levels of gene cluster members spanning an entire continuous region (44–46). Consistent with these findings, we observed that increased DNA methylation at cg20401567 correlated with reduced expression of *HOXB* gene cluster members and *HOXB-AS* members. *HOX* gene clusters are large superfamilies of genes whose members play fundamental roles in cell development (47). Boimel et al. reported that *HOXB2* knockdown promoted primary tumor growth in mammary adenocarcinoma cell lines, suggesting that in this context, it functions as a tumor suppressor (48). Intriguingly, there is evidence that in addition to the *HOX* gene coding regions, the *cis*-regulatory regions, including intergenic and antisense transcribed regions, provide an extra layer of regulation of *HOX* expression (49). We observed that the genomic region around cg20401567 is highly conserved and enriched with various *cis*-regulatory elements in the integrative functional annotation map, which provides direct evidence supporting this model. This CpG locus is not in a gene promoter and its methylation affects at least 7 genes in the *HOXB* cluster spreading over 50 kb, thus, its mechanism of gene silencing is unlikely to be the same as canonical promoter repression (50). The current study aimed to provide further insights into why Black women are more likely than White women to be diagnosed with aggressive breast cancer, particularly ER- breast cancer. Reduced expression of *HOXB2* in breast tumors from Black versus White women represents a novel molecular feature which may be linked to racial differences in breast tumor biology and outcomes. Moreover, our findings support a novel regulatory site for *HOXB2* activity, which could be a potential therapeutic target in breast cancer treatment.

*FOXA1* is an important transcription factor playing crucial roles in mammary gland development. We previously reported a *FOXA1* raDML (cg04932551), located in the gene body, which is annotated as a poised promoter. This site was hypermethylated in tumors from Black versus White women, and its methylation was inversely associated with *FOXA1* RNA and protein expression in ER- tumors among Black women (12, 51). We further found that methylation and expression of *FOXA1* is associated with parity and breastfeeding, suggesting a potential mechanism that links these reproductive exposures with ER- breast cancer among Black women (12). In the current study, we identified another raDML (cg12212453), located at 120 bp downstream of *FOXA1*, which was negatively correlated with *FOXA1* expression. Our analysis did not show associations (data not shown) of cg12212453 methylation with reproductive factors, suggesting it is less likely to mediate effects of reproductive exposures on cancer predisposition. Nevertheless, this CpG might represent a novel regulatory site for *FOXA1* expression and warrants further investigations.

*SLC39A11* belongs to a member of a large family of membrane transport proteins participating in wide range of physiological processes (52). Significantly enhanced *SLC39A* family of proteins are expressed in multiple malignances including colorectal cancer, breast cancer, and esophageal cancer (53, 54). We observed a higher methylation of *SLC39A11* (cg05322837) correlated with lower *SLC39A11* expression levels in tumors from Black compared to that in White, implicating that differences in methylation and expression of *SLC39A11* may contribute to breast cancer racial disparities. Intriguingly, methylation at cg05322837 was positively correlated with expression of two overlapping lncRNAs, *LOC102723517* and *LINC01152*, as well as *SOX9-AS1*. The cg05322837 is in a putative enhancer region approximately 500 kb upstream of *SLC39A11* and about 100kb downstream of the two lncRNAs, thus it is possible that *SLC39A11* transcription is targeted by *LOC102723517* and *LINC01152*, with its expression down regulated by methylation at cg05322837.

*SCUBE2* is another gene with its expression level positively correlated with DNA methylation at a CpG site (cg05199874) approximately 500kb upstream. It exhibited differential expression by race both in our study population and the TCGA breast cancer cohort. *SCUBE2* was reported to work synergistically with *FOXA1* as a novel breast-tumor suppressor, driving the reversal of epithelial–mesenchymal transition (EMT) (36). *SCUBE2* transcription was epigenetically inactivated by recruitment of DNA methyltransferase 1 onto its CpG islands during EMT while the exact CpGs remain unclear (36). We speculated that the downregulation of *SCUBE2* in concert with *FOXA1* is part of the EMT program that plays important roles in modulating breast-cancer cell migration and invasion. Our results implicated that EMT could also contribute to racial differences in ER- breast cancer predisposition and revealed that specific *cis* elements through which DNA methylation may influence two key regulators of EMT. Future studies are warranted to investigate this intriguing finding.

Transcription factors impact gene expression through binding to either positive (such as promoter and enhancer) or negative (such as silencer and insulator) regulatory elements under certain chromatin structure, which is drastically affected by epigenetic modifications including DNA methylation. Thus far, dysregulated DNA methylation in promoter region has attracted much of the research attention. How DNA methylation in other genome regions affects gene expression remains largely unknown. In our study, while the same CpG sites correlated with two genes’ expression levels in diverse direction, we also observed three CpG sites residing a small CpG island correlated with increased expression of the same genes, indicating these three consecutive CpG sites could belongs to the same regulatory element. The multiple correlation patterns between DNA methylation and gene expression suggested complex regulatory mechanisms of these *cis*-elements.

In summary, unlike previous studies that focused primarily on CpGs mapped to protein-coding genes, this study focuses on aberrant DML located in the noncoding genome regions, with the aim of interrogating biological mechanisms underlying the observed racial differences of high risk of ER- breast tumors in Black women compared to White women. We identified several important genes, being implicated in breast cancer pathology, with their expression correlated with aberrant DNA methylation of CpGs located in noncoding genome regions. The functional potentiality of these aberrantly methylated CpGs were further examined through integrative, molecular feature annotations. These results were subsequently validated in the independent TCGA breast cancer cohort. Our results provide new insights into the contribution of aberrant DNA methylation within the non-protein coding region to breast cancer racial disparities. Further experimental validations will be warranted to confirm these findings in future studies.

## Data availability statement

The datasets presented in this study can be found in online repositories. The names of the repository/repositories and accession number(s) can be found in the article/**Supplementary Material**.

## Ethics statement

This study was approved by the institutional review boards of Roswell Park Comprehensive Cancer Center. The patients/participants provided their written informed consent to participate in this study.

## Author contributions

CA, ZG and MH conceived and supervised the projects. QH, TK, and SL finished acquisition of all data. JC and QH did all statistical analysis and wrote the original draft. JC, MH, ZG, and CA reviewed and edited the manuscript. All authors contributed to the article and approved the submitted version.

## Supplementary material

Supplementary Figure 1 Validation of raDMLs identified in current (WCHS) study using the TCGA breast cancer cohort. Scatter plot of delta beta value of raDMLs from WCHS (X-axis) versus TCGA (Y-axis). Fifty-nine out of the 96 raDMLs were available in the TCGA breast cancer dataset. Fifty-eight raDMLs identified in both data, with a consistent direction of methylation changes (delta beta) and FDR-adjusted *P*<0.05, were plotted as black dots, whereas one CpG site showing an inconsistent methylation change was labeled in red. Cg20401567 was highlighted and labeled in green.

Supplementary Figure 2 An integrative annotation showing molecular features of the genomic regions of Cg20401567/*HOXB/HOXB-AS* gene clusters. From top to bottom, the tracks showing: *HOXB* and *HOXB-AS* gene clusters; the Cg20401567 site highlighted in vertical cyan line; measurements of evolutionary conservation from alignments of 100 vertebrate species; conserved transcriptional binding sites; the hypersensitive DNaseI sites profile of HMEC and MCF cell lines of the ENCODE project; the HMEC ChromHMM tracks indicating putative active (bright red) promoters, strong enhancer (orange), strong transcript (green), as well as putative weak enhancers (yellow); histone modifications of H3K27ac, H3K4me1/3 in various breast cell types.
