# Supplementary figures and images for: DNA methylation differences in noncoding regions in ER negative breast tumors between Black and White women
Source: Front Oncol. 2023 May 24;13:1167815. doi: 10.3389/fonc.2023.1167815 (PMC10244512; doi:10.3389/fonc.2023.1167815)

Figure S1.

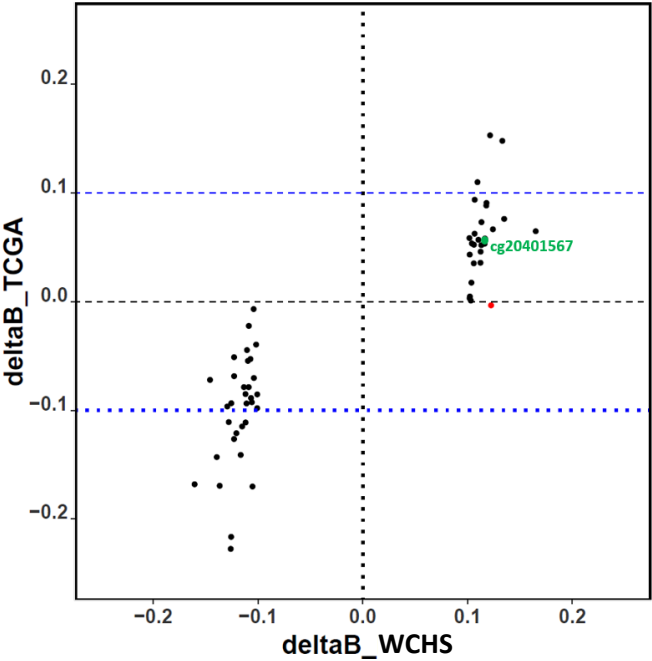

Figure S2.

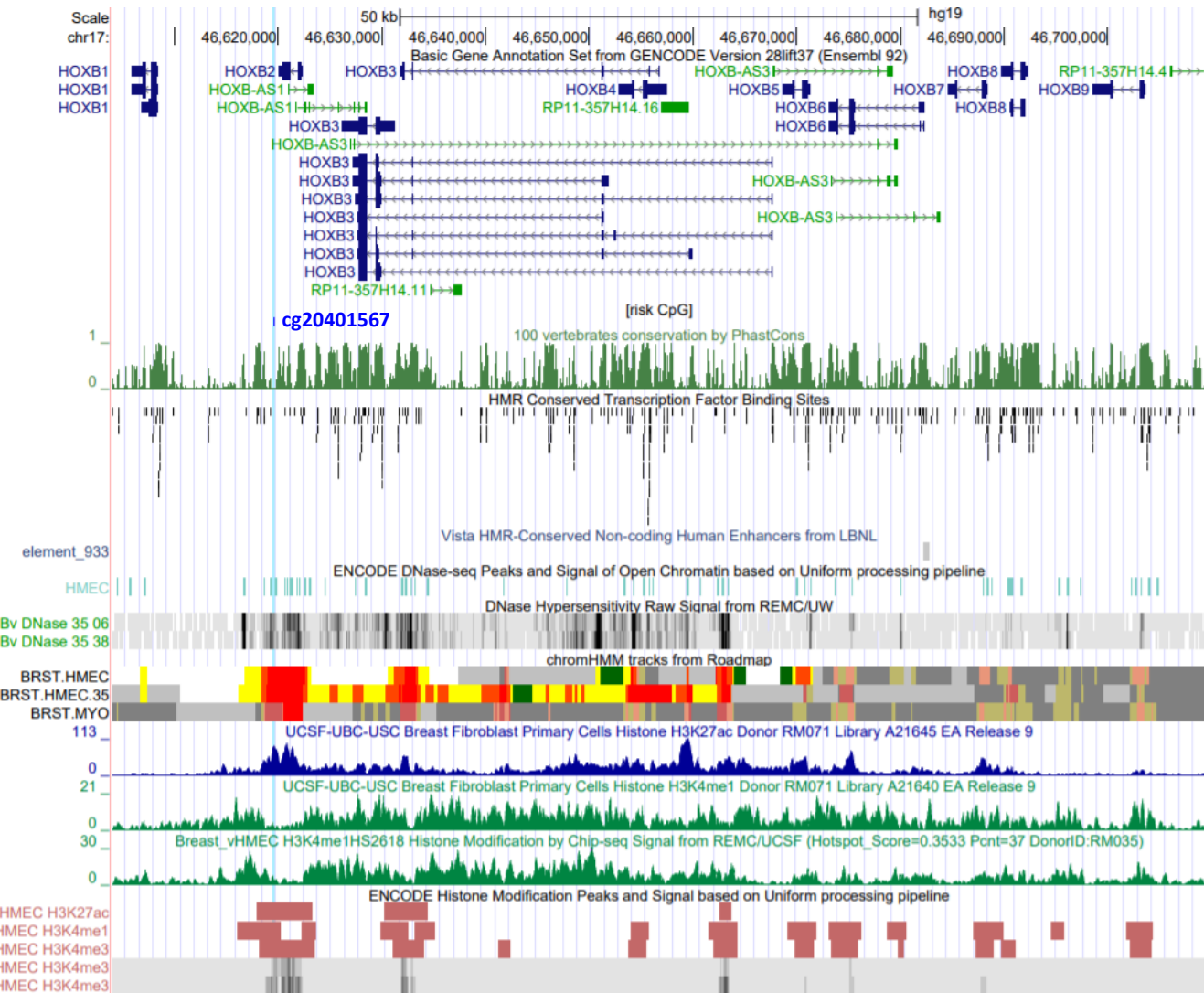

Supplement: Supplementary Figure 1 — Validation of raDMLs identified in current (WCHS) study using the TCGA breast cancer cohort. Scatter plot of delta beta value of raDMLs from WCHS (X-axis) versus TCGA (Y-axis). Fifty-nine out of the 96 raDMLs were available in the TCGA breast cancer dataset. Fifty-eight raDMLs identified in both data, with a consistent direction of methylation changes (delta beta) and FDR-adjusted P<0.05, were plotted as black dots, whereas one CpG site showing an inconsistent methylation change was labeled in red. Cg20401567 was highlighted and labeled in green. [file DataSheet_1.pdf]
