# Supplementary material for: DNA methylation differences in noncoding regions in ER negative breast tumors between Black and White women
Source: Front Oncol. 2023 May 24;13:1167815. doi: 10.3389/fonc.2023.1167815 (PMC10244512; doi:10.3389/fonc.2023.1167815)
Supplement: Supplementary file 2 [file Table_1.docx]

**Table S1**. Demographic and tumor characteristics of study participants from the WCHS cohort.

|  |  | Black (n=368) | White (n=326) | *P^1^* |
| --- | --- | --- | --- | --- |
| Age at diagnosis | Mean ± std | 51.7±10.7 | 52.3±10.3 | 0.51 |
|  | 45 below | 93 (25.3) | 81 (24.9) | 0.97 |
|  | 45-55 | 136 (37.0) | 114 (35.0) |  |
|  | 56-65 | 111 (30.2) | 100 (30.7) |  |
|  | 66-75 | 31 (8.4) | 31 (9.5) |  |
| BMI (kg/m^2^) |  |  |  |  |
|  | Mean ± std | 30.92±6.9 | 26.84±5.7 | <0.001 |
|  | < 25.0 (normal) | 57 (18.3) | 124 (44.8) | <0.001 |
|  | 25-29.9 (overweight) | 101 (32.4) | 78 (28.2) |  |
|  | >= 30.0 (obesity) | 154 (49.4) | 75 (27.1) |  |
| Menopausal status |  |  |  |  |
|  | Yes | 161 (49.5) | 152 (52.8) | 0.47 |
|  | No | 164 (50.5) | 136 (47.2) |  |
| Family history |  |  |  |  |
|  | Yes | 272 (84.7) | 221 (78.4) | 0.045 |
|  | No | 49 (15.3) | 61 (21.6) |  |
| ER status |  |  |  |  |
|  | Negative | 107 (29.8) | 53 (16.3) | <0.001 |
|  | Positive | 261 (70.2) | 273 (83.7) |  |

^1^ Based on chi-square test for categorical variables and t test for continuous variables.

**Table S2.** Ninety-six raDMLs within non-protein coding regions in ER- tumors by race.

| **CpG** | **chr** | **mapInfo_hg19** | **mapInfo_hg38** | **deltaBeta^a^** | **padj^b^** |
| --- | --- | --- | --- | --- | --- |
| cg00428457 | 2 | 119887680 | 119130104 | 0.12 | 4.99E-04 |
| cg00806644 | 10 | 126889815 | 125201246 | -0.12 | 2.75E-04 |
| cg00924575 | 5 | 76474673 | 77178848 | -0.13 | 4.11E-05 |
| cg01013171 | 14 | 75716369 | 75249666 | -0.11 | 1.04E-04 |
| cg02096220 | 4 | 129212177 | 128291022 | 0.11 | 3.15E-04 |
| cg02264082 | 3 | 130235850 | 130517006 | 0.11 | 1.47E-03 |
| cg02291164 | 14 | 59296302 | 58829584 | -0.10 | 6.53E-05 |
| cg02464458 | 8 | 1203186 | 1253186 | -0.11 | 1.24E-03 |
| cg02772171 | 5 | 171054642 | 171627638 | 0.21 | 7.08E-07 |
| cg03071808 | 2 | 242908131 | 241965980 | -0.11 | 7.52E-04 |
| cg03518729 | 1 | 235147744 | 235011997 | -0.12 | 2.68E-03 |
| cg03555299 | 2 | 206830681 | 205965957 | 0.11 | 9.05E-04 |
| cg03702477 | 5 | 152374989 | 152995429 | -0.11 | 3.61E-04 |
| cg03988092 | 4 | 190236439 | 189315285 | 0.12 | 1.22E-04 |
| cg04220930 | 8 | 52814681 | 51902121 | -0.11 | 7.29E-04 |
| cg04553707 | 17 | 53922670 | 55845309 | 0.12 | 6.28E-04 |
| cg05199874 | 11 | 9623178 | 9601631 | -0.13 | 4.72E-04 |
| cg05322837 | 17 | 70147783 | 72151642 | 0.13 | 1.19E-04 |
| cg05393297 | 12 | 53359155 | 52965371 | -0.13 | 4.92E-05 |
| cg05547895 | 3 | 196705855 | 196978984 | -0.10 | 7.65E-04 |
| cg05997655 | 16 | 53544321 | 53510409 | -0.14 | 4.60E-04 |
| cg06093861 | 7 | 75780412 | 76151094 | -0.20 | 1.22E-10 |
| cg06611487 | 5 | 115284110 | 115948413 | 0.12 | 5.64E-05 |
| cg06715628 | 12 | 52482292 | 52088508 | -0.16 | 3.36E-08 |
| cg07125829 | 11 | 45822831 | 45801280 | -0.10 | 7.90E-02 |
| cg07379055 | 19 | 12305553 | 12194738 | 0.11 | 2.41E-04 |
| cg07502444 | 19 | 57682795 | 57171427 | 0.11 | 4.55E-05 |
| cg07870920 | 4 | 121569769 | 120648614 | 0.11 | 1.91E-02 |
| cg08496483 | 5 | 55863072 | 56567245 | -0.13 | 3.49E-03 |
| cg08636385 | 6 | 71816668 | 71106965 | -0.11 | 1.64E-03 |
| cg08750459 | 17 | 6558815 | 6655496 | 0.11 | 4.55E-04 |
| cg08782356 | 5 | 33092857 | 33092751 | -0.13 | 4.39E-04 |
| cg09351263 | 16 | 85864047 | 85830441 | 0.36 | 1.22E-10 |
| cg09372486 | 19 | 31845489 | 31354583 | -0.11 | 8.16E-05 |
| cg09455823 | 2 | 201606912 | 200742189 | 0.12 | 3.96E-05 |
| cg09704168 | 17 | 32751554 | 34424535 | 0.17 | 4.09E-07 |
| cg10070864 | 15 | 57664490 | 57372292 | -0.16 | 2.62E-10 |
| cg10321395 | 11 | 1107803 | 1113895 | -0.14 | 5.94E-05 |
| cg10578777 | 12 | 7781093 | 7628497 | -0.14 | 3.47E-06 |
| cg10625247 | 2 | 221063983 | 220199262 | 0.11 | 2.39E-04 |
| cg10781513 | 3 | 193921641 | 194203852 | -0.15 | 4.39E-06 |
| cg11072794 | 19 | 388349 | 388349 | -0.13 | 4.55E-05 |
| cg11235391 | 8 | 49343007 | 48430447 | 0.10 | 6.60E-03 |
| cg11497372 | 6 | 27513092 | 27545313 | 0.10 | 4.03E-03 |
| cg12096354 | 15 | 70816640 | 70524301 | -0.15 | 1.47E-03 |
| cg12212453 | 14 | 38058639 | 37589434 | 0.12 | 2.28E-03 |
| cg12361223 | 3 | 147089362 | 147371575 | 0.10 | 1.47E-03 |
| cg12821539 | 13 | 100642775 | 99990521 | 0.11 | 4.15E-03 |
| cg13380890 | 12 | 76414534 | 76020754 | 0.10 | 1.63E-02 |
| cg13692134 | 5 | 74350132 | 75054307 | 0.11 | 2.40E-07 |
| cg14795227 | 2 | 239565255 | 238656614 | -0.16 | 3.41E-06 |
| cg15637286 | 19 | 31845176 | 31354270 | -0.11 | 3.32E-04 |
| cg15671450 | 6 | 29895116 | 29927339 | -0.11 | 1.68E-04 |
| cg16301894 | 4 | 129389744 | 128468589 | 0.10 | 2.87E-03 |
| cg16329197 | 12 | 53359506 | 52965722 | -0.13 | 3.58E-04 |
| cg16426479 | 5 | 43020566 | 43020464 | -0.12 | 1.65E-04 |
| cg16634718 | 10 | 52496341 | 50736581 | -0.11 | 3.99E-05 |
| cg17148876 | 12 | 95730175 | 95336399 | 0.12 | 1.21E-05 |
| cg17916960 | 15 | 79447300 | 79154958 | 0.11 | 3.64E-04 |
| cg17982497 | 15 | 96906859 | 96363630 | -0.12 | 2.12E-04 |
| cg18096722 | 8 | 70947218 | 70034983 | 0.10 | 7.94E-03 |
| cg18272538 | 1 | 210425852 | 210252507 | -0.10 | 2.45E-04 |
| cg18514595 | 22 | 49579968 | 49184042 | 0.18 | 4.89E-04 |
| cg19034132 | 10 | 75692227 | 73932469 | -0.11 | 4.75E-05 |
| cg19214707 | 7 | 3157722 | 3118088 | -0.10 | 4.17E-03 |
| cg19318364 | 6 | 55774999 | 55910201 | -0.11 | 6.28E-04 |
| cg19848924 | 10 | 34344580 | 34055652 | -0.28 | 1.66E-10 |
| cg20222562 | 6 | 113993995 | 113672793 | 0.12 | 6.82E-05 |
| cg20316758 | 13 | 106797152 | 106144803 | -0.11 | 1.17E-03 |
| cg20401567 | 17 | 46619555 | 48542193 | 0.12 | 4.30E-04 |
| cg20432671 | 1 | 167682922 | 167713685 | -0.11 | 2.00E-03 |
| cg20593831 | 2 | 119532492 | 118774916 | 0.10 | 6.46E-02 |
| cg21028156 | X | 2743660 | 2825619 | 0.37 | 5.88E-11 |
| cg21399203 | 4 | 57623913 | 56757747 | -0.14 | 3.09E-04 |
| cg21406075 | 15 | 96906354 | 96363125 | -0.11 | 6.25E-05 |
| cg22283083 | 2 | 53160678 | 52933540 | -0.11 | 3.97E-04 |
| cg22491409 | 12 | 125198295 | 124713749 | -0.12 | 2.53E-04 |
| cg22676075 | 6 | 135203613 | 134882475 | -0.12 | 1.51E-03 |
| cg22851875 | 5 | 65888284 | 66592456 | -0.31 | 6.18E-06 |
| cg23126094 | 10 | 3235857 | 3193665 | -0.12 | 3.38E-03 |
| cg23363182 | 2 | 181467187 | 180602460 | -0.12 | 2.67E-08 |
| cg23493412 | 17 | 63491688 | 65495570 | -0.10 | 1.28E-02 |
| cg24114813 | 5 | 172128837 | 172701834 | -0.13 | 2.37E-04 |
| cg24158844 | 1 | 1079561 | 1144181 | -0.10 | 9.59E-06 |
| cg24694833 | 22 | 43168851 | 42772845 | 0.15 | 1.02E-04 |
| cg24935598 | 3 | 193921489 | 194203700 | -0.11 | 4.36E-05 |
| cg25025879 | 12 | 53359317 | 52965533 | -0.11 | 4.00E-03 |
| cg25101657 | 17 | 32751628 | 34424609 | 0.13 | 6.50E-06 |
| cg25303761 | 1 | 31256028 | 30783181 | -0.13 | 9.21E-04 |
| cg25570913 | 13 | 112759893 | 112105579 | 0.11 | 1.39E-02 |
| cg25828445 | 12 | 7781288 | 7628692 | -0.16 | 1.13E-07 |
| cg26099045 | 2 | 64291800 | 64064666 | 0.11 | 8.14E-04 |
| cg26473818 | 11 | 13945715 | 13924168 | -0.11 | 1.20E-03 |
| cg26804772 | 1 | 68084465 | 67618782 | 0.10 | 5.81E-04 |
| cg27093944 | 2 | 242908218 | 241966067 | -0.12 | 3.97E-04 |
| cg27539046 | 1 | 32451252 | 31985651 | -0.10 | 1.39E-03 |

^a^ DNA methylation difference (delta Beta) in ER- tumors between Black vs. White women

^b^ Adjusted p-value for the DNA methylation differences between races

**Table S3.** Thirty-nine CpG/gene pairs remained significantly correlated between DNA

methylation and gene expression within ER- breast cancer.

| **CpG** | **Gene** | **deltaBeta^a^** | **padj^b^** | **Rho^c^** | **padj_rho^d^** |
| --- | --- | --- | --- | --- | --- |
| cg20401567 | *HOXB2* | 0.12 | 4.30E-04 | -0.74 | 2.87E-06 |
| cg12212453 | *FOXA1* | 0.12 | 2.28E-03 | -0.67 | 1.06E-04 |
| cg05322837 | *LOC102723517* | 0.13 | 1.19E-04 | 0.65 | 2.28E-04 |
| cg12821539 | *ZIC5* | 0.11 | 4.15E-03 | 0.62 | 7.36E-04 |
| cg20401567 | *HOXB3* | 0.12 | 4.30E-04 | -0.62 | 7.36E-04 |
| cg05322837 | *SLC39A11* | 0.13 | 1.19E-04 | -0.61 | 7.96E-04 |
| cg05199874 | *TMEM41B* | -0.13 | 4.72E-04 | 0.58 | 2.47E-03 |
| cg05199874 | *RNF141* | -0.13 | 4.72E-04 | 0.58 | 2.47E-03 |
| cg05322837 | *LINC01152* | 0.13 | 1.19E-04 | 0.58 | 3.04E-03 |
| cg05199874 | *SCUBE2* | -0.13 | 4.72E-04 | 0.55 | 8.26E-03 |
| cg03071808 | *D2HGDH* | -0.11 | 7.52E-04 | 0.54 | 1.29E-02 |
| cg05322837 | *SOX9-AS1* | 0.13 | 1.19E-04 | 0.53 | 1.55E-02 |
| cg27539046 | *IQCC* | -0.10 | 1.39E-03 | 0.53 | 1.55E-02 |
| cg00428457 | *EN1* | 0.12 | 4.99E-04 | 0.52 | 1.56E-02 |
| cg05393297 | *ACVR1B* | -0.13 | 4.92E-05 | 0.52 | 1.56E-02 |
| cg07502444 | *MIMT1* | 0.11 | 4.55E-05 | 0.52 | 1.56E-02 |
| cg07870920 | *QRFPR* | 0.11 | 1.91E-02 | 0.52 | 1.56E-02 |
| cg09455823 | *SGOL2* | 0.12 | 3.96E-05 | 0.52 | 1.56E-02 |
| cg10625247 | *GMPPA* | 0.11 | 2.39E-04 | -0.51 | 1.56E-02 |
| cg23363182 | *ITGA4* | -0.12 | 2.67E-08 | -0.52 | 1.56E-02 |
| cg16329197 | *ACVR1B* | -0.13 | 3.58E-04 | 0.49 | 2.70E-02 |
| cg20401567 | *HOXB-AS1* | 0.12 | 4.30E-04 | -0.49 | 2.70E-02 |
| cg24114813 | *ERGIC1* | -0.13 | 2.37E-04 | 0.50 | 2.70E-02 |
| cg00428457 | *EPB41L5* | 0.12 | 4.99E-04 | -0.49 | 2.84E-02 |
| cg02291164 | *C14orf37* | -0.10 | 6.53E-05 | 0.49 | 3.13E-02 |
| cg02291164 | *PSMA3* | -0.10 | 6.53E-05 | -0.48 | 3.13E-02 |
| cg12821539 | *ZIC2* | 0.11 | 4.15E-03 | 0.48 | 3.13E-02 |
| cg25025879 | *ACVR1B* | -0.11 | 4.00E-03 | 0.49 | 3.13E-02 |
| cg19034132 | *P4HA1* | -0.11 | 4.75E-05 | 0.48 | 3.44E-02 |
| cg17148876 | *HAL* | 0.12 | 1.21E-05 | 0.48 | 3.70E-02 |
| cg11235391 | *EFCAB1* | 0.10 | 6.60E-03 | 0.47 | 3.91E-02 |
| cg20401567 | *HOXB5* | 0.12 | 4.30E-04 | -0.47 | 3.91E-02 |
| cg02772171 | *GABRP* | 0.21 | 7.08E-07 | 0.47 | 4.37E-02 |
| cg12212453 | *MIPOL1* | 0.12 | 2.28E-03 | -0.47 | 4.37E-02 |
| cg20401567 | *COPZ2* | 0.12 | 4.30E-04 | -0.47 | 4.37E-02 |
| cg27093944 | *D2HGDH* | -0.12 | 3.97E-04 | 0.47 | 4.37E-02 |
| cg05393297 | *LOC283335* | -0.13 | 4.92E-05 | 0.46 | 4.54E-02 |
| cg20401567 | *HOXB-AS3* | 0.12 | 4.30E-04 | -0.46 | 4.73E-02 |
| cg20401567 | *HOXB4* | 0.12 | 4.30E-04 | -0.46 | 4.95E-02 |

^a^ DNA methylation difference (delta Beta) in ER- tumors between Black vs. White women

^b^ Adjusted p-value for DNA methylation comparisons between races

^c^ Spearman correlation coefficient

^d^ Adjusted p-value for DNA methylation-gene expression correlation

**Table S4.** Correlation validation analysis between DNA methylation and gene expression using the

TCGA breast cancer cohort.

| **Probe** | **Gene** | **Coeff_Cor.x ^a^** | **padj_Cor.x ^b^** | **Coeff_Cor.y ^c^** | **padj_Cor.y ^d^** | **Consistency^e^** |
| --- | --- | --- | --- | --- | --- | --- |
| cg00428457 | EN1 | 0.52 | 1.56E-02 | 0.66 | 1.10E-26 | 1 |
| cg00428457 | EPB41L5 | -0.49 | 2.84E-02 | -0.62 | 4.94E-23 | 1 |
| cg02291164 | C14orf37 | 0.49 | 3.13E-02 | 0.21 | 1.11E-02 | 1 |
| cg02291164 | PSMA3 | -0.48 | 3.13E-02 | -0.11 | 2.00E-01 | 2 |
| cg05199874 | RNF141 | 0.58 | 2.47E-03 | 0.50 | 9.59E-14 | 1 |
| cg05199874 | SCUBE2 | 0.55 | 8.26E-03 | 0.60 | 1.10E-20 | 1 |
| cg05199874 | TMEM41B | 0.58 | 2.47E-03 | 0.41 | 9.56E-09 | 1 |
| cg05322837 | SLC39A11 | -0.61 | 7.96E-04 | -0.52 | 1.03E-14 | 1 |
| cg05393297 | ACVR1B | 0.52 | 1.56E-02 | 0.26 | 7.27E-04 | 1 |
| cg07502444 | MIMT1 | 0.52 | 1.56E-02 | -0.10 | 2.64E-01 | 2 |
| cg09455823 | SGOL2 | 0.52 | 1.56E-02 | 0.23 | 3.88E-03 | 1 |
| cg10625247 | GMPPA | -0.51 | 1.56E-02 | -0.12 | 1.92E-01 | 2 |
| cg11235391 | EFCAB1 | 0.47 | 3.91E-02 | -0.01 | 9.15E-01 | 2 |
| cg12212453 | FOXA1 | -0.67 | 1.06E-04 | -0.74 | 1.17E-35 | 1 |
| cg12212453 | MIPOL1 | -0.47 | 4.37E-02 | -0.34 | 7.47E-06 | 1 |
| cg12821539 | ZIC2 | 0.48 | 3.13E-02 | 0.42 | 2.31E-09 | 1 |
| cg12821539 | ZIC5 | 0.62 | 7.36E-04 | 0.38 | 1.37E-07 | 1 |
| cg16329197 | ACVR1B | 0.49 | 2.70E-02 | 0.22 | 6.00E-03 | 1 |
| cg17148876 | HAL | 0.48 | 3.70E-02 | 0.10 | 2.87E-01 | 2 |
| cg19034132 | P4HA1 | 0.48 | 3.44E-02 | 0.14 | 1.13E-01 | 2 |
| cg20401567 | COPZ2 | -0.47 | 4.37E-02 | -0.22 | 6.60E-03 | 1 |
| cg20401567 | HOXB2 | -0.74 | 2.87E-06 | -0.75 | 5.10E-39 | 1 |
| cg20401567 | HOXB3 | -0.62 | 7.36E-04 | -0.66 | 1.05E-26 | 1 |
| cg20401567 | HOXB4 | -0.46 | 4.95E-02 | -0.58 | 1.66E-19 | 1 |
| cg20401567 | HOXB5 | -0.47 | 3.91E-02 | -0.49 | 7.60E-13 | 1 |
| cg23363182 | ITGA4 | -0.52 | 1.56E-02 | -0.15 | 8.24E-02 | 2 |
| cg25025879 | ACVR1B | 0.49 | 3.13E-02 | 0.24 | 3.21E-03 | 1 |
| cg27539046 | IQCC | 0.53 | 1.55E-02 | -0.09 | 3.43E-01 | 2 |

^a^ Coeff_Cor.x Spearman correlation coefficient between DNA methylation and RNA expression levels in the WCHS cohort.

^b^ padj_Cor.x Adjusted p-value for the correlation coefficient in the WCHS cohort.

^c^ Coeff_Cor.y Spearman correlation coefficient between DNA methylation and RNA expression levels in the TCGA cohort.

^d^ padj_Cor.y Adjusted p-value for the correlation coefficient in the TCGA cohort.

^e^ Consistency of correlation analysis in the WCHS and the TCGA breast cancer cohort. "1" denotes consistency and "2" denotes inconsistency of correlation coefficients between the two cohorts.
